# Sulfated and Phosphorylated Agarose as Biomaterials for a Biomimetic Paradigm for FGF-2 Release

**DOI:** 10.3390/biomimetics10010012

**Published:** 2024-12-30

**Authors:** Aurelien Forget, V. Prasad Shastri

**Affiliations:** 1 Institute for Macromolecular Chemistry, Stefan-Meier-Strasse 31, 79104 Freiburg, Germany; aurelien.forget@gmail.com; 2 BIOSS, Centre for Biological Signalling, Schanzelstrasse 18, 79104 Freiburg, Germany

**Keywords:** biomimetic, angiogenesis, growth factors, controlled release, regenerative medicine

## Abstract

Cardiovascular diseases such as myocardial infarction or limb ischemia are characterized by regression of blood vessels. Local delivery of growth factors (GFs) involved in angiogenesis such as fibroblast blast growth factor-2 (FGF-2) has been shown to trigger collateral neovasculature and might lead to a therapeutic strategy. In vivo, heparin, a sulfated polysaccharide present in abundance in the extracellular matrix (ECM), has been shown to function as a local reservoir for FGF-2 by binding FGF-2 and other morphogens and it plays a role in the evolution of GF gradients. To access injectable biomaterials that can mimic such natural electrostatic interactions between soluble signals and macromolecules and mechanically tunable environments, the backbone of agarose, a thermogelling marine–algae-derived polysaccharide, was modified with sulfate, phosphate, and carboxylic moieties and the interaction and release of FGF-2 from these functionalized hydrogels was assessed by ELISA in vitro and CAM assay in ovo. Our findings show that FGF-2 remains active after release, and FGF-2 release profiles can be influenced by sulfated and phosphorylated agarose, and in turn, promote varied blood vessel formation kinetics. These modified agaroses offer a simple approach to mimicking electrostatic interactions experienced by GFs in the extracellular environment and provide a platform to probe the role of these interactions in the modulation of growth factor activity and may find utility as an injectable gel for promoting angiogenesis and as bioinks in 3D bioprinting.

## 1. Introduction

The extracellular matrix (ECM), derived from proteins and polysaccharides synthesized by cells, provides structural support and presents molecules to which cells can attach [1]. The ECM polysaccharides namely, hyaluronic acid, heparin sulfate, keratan sulfate, dermatan sulfate, and chondroitin sulfate are highly sulfated and occur as protein conjugates (proteoglycans) except hyaluronic acid, which is also carboxylated [1]. Proteoglycans are found in various tissues, including the brain [2], cartilage [3], and skin [4], as well as tissues undergoing repair, such as exudates from partial-thickness skin excision wounds [5]. In addition to providing osmotic activity, ECM polysaccharides, being highly negatively charged, interact with positively charged domains in several proteins through electrostatic interactions. Such interactions result in the sequestration of growth factors (GFs) and morphogens, leading to local reservoirs of signaling molecules [6] and the generation of GF and morphogen gradients, which are critical during development, organogenesis, cellular polarization, migration, proliferation, and angiogenesis—the formation of new blood vessels [7,8]. One class of ECM molecules identified as capable of immobilizing soluble factors along its backbone are sulfated polysaccharides [9]. Indeed, sulfated charged moieties present on the heparin backbone have been shown to play a role in the immobilization of growth factors such as transforming growth factor-beta [10,11] and basic fibroblast-derived growth factor-2 (FGF-2) [12] through electrostatic interactions [7,13] and confer protection to both acid and basic FGF from inactivation [14]. The highly charged polysaccharides in the ECM also play a prominent role in tissue hydration and confer hydrogel-like characteristics to tissue. As a result, hydrogels have been extensively explored in engineering cellular environments [15]. 

In addition to soluble biological signals, the mechanical properties of the cell, the ECM, and the tissue have been shown to play a critical role in cell fate and tissue homeostasis [16,17]. Since the early 2000s, several studies have elaborated on the role of tissue stiffness in both normal and cancer tissue [18,19]. Interestingly, cell-surface proteoglycans—glycocalyx, have been shown to contribute to mechanobiology and mediate cancer progression [20]. Given these findings, hydrogels with tunable mechanical properties that can emulate biomimetic interactions with growth factors can aid in investigating the role of tissue mechanics in GF presentation and signaling. In addition to synthetic polymers, proteins, and peptides, marine–algae-derived polysaccharides can also form hydrogels [21] and can, therefore, be exploited for growth factor delivery if they possess the appropriate charge characteristics, i.e., anion and density of anions [22]. While carrageenans, which are extracted from red edible seaweed, are naturally sulfated, most marine polysaccharides, such as alginate and agarose which form hydrogels through ionic and physical crosslinking mechanisms, respectively, have carboxylic acid along their backbone [21]. Therefore, expanding the repertoire of negative charges along the polysaccharide backbone can further its utilization in the engineering of bespoke biomimetic materials for regenerative therapies.

We have recently shown that carboxylation of the agarose backbone (carboxylated agarose (CA)) can induce a switch in the secondary structure of the agarose chain from an alpha helix to a beta-sheet [23], resulting in hydrogels with tunable mechanical properties in the range of 10 Pa–10^4^ kPa [24]. Using CA hydrogels with mechanical properties similar to granulation tissue and blot clot, it has been shown in a seminal study that the activation of RGD signaling in endothelial cells (ECs) within these environments can synergistically induce the apical-basal polarization of ECs and their organization into tubular structures in vitro [24], and in vivo, promote the recruitment of mechanosensing Piezo-1 positive monocytes leading to the stabilization of neovasculature through sprouting angiogenesis [25]. Carboxylated agarose has also been extensively explored as bioinks in 3D bioprinting [26,27,28], as an extrudable cell carrier [29] and drug delivery matrix [30]. Building on these advancements, in this study, we investigated the release kinetics of FGF-2 from agarose modified with different negatively charged moieties found in natural polysaccharides [31,32,33], namely, carboxylic acid, sulfate, and phosphate. Specifically, we aimed to elucidate the impact of the backbone modifications on the polysaccharide secondary structure and how this influences hydrogel structure, fiber organization, rheological properties, and the sequestration and release of FGF-2 using two functional assays: (1) enzyme-linked immunosorbent assay (ELISA) and (2) the in ovo chorioallantoic membrane (CAM) angiogenesis assay. FGF-2 was chosen as a model GF as it has been extensively investigated as a therapeutic molecule due to its prominent role in cell proliferation and sprouting angiogenesis [34]. Our findings suggest that backbone charge impacts not only FGF-2 release behavior but also the formation of blood vessels in the CAM assay. Since the modified agaroses retain their gel-forming properties, they provide a unique platform to investigate the synergy between matrix mechanics and GF signaling in cell differentiation and organization.

## 2. Materials and Methods

### 2.1. Material

Agarose (type I low melting) was obtained from Merck (Darmstadt, Germany, DE, M_w_140,000 g/mol, Ð 1.5-2). TEMPO (((2,2,6,6-Tetramethylpiperidin-1-yl) oxyl), NaOCl (sodium hypochlorite), NaBH4 (sodium borohydride), NaBr (sodium bromide), and KBr (potassium bromide) were used as received (Sigma Aldrich, Hamburg, Germany). Dialysis membranes with MWCO (Molecular Weight Cut Off) 12–14 kDa and 3.5 kDa (Spectrum Laboratories, Gardena, CA, USA) were purchased from Carl Roth (Karlsruhe, Germany). Freeze drying was carried out on a Beta 2-8 LD plus from Christ (Osterode am Harz, Germany). Chlorosulfonic acid was obtained from Sigma Aldrich (Germany) and used as received. Formamide, urea, and phosphoric acid were obtained from Sigma Aldrich (Germany) and used as received. Methanol, pyridine, and ethanol were obtained from Sigma Aldrich (Germany) and used as received except when stated otherwise. Solutions of 0.5 M NaOH (sodium hydroxide) and 5 M HCl (hydrochloric acid) were freshly prepared before each synthesis. Water was purified with a Milli-Q Reference filtration system (Millipore, Darmstadt, Germany) and then sterilized by autoclaving (Systec, DX 150, Systec, Linden, Germany) using the liquid cycle (121 °C, 220 kPa for 15 min). FGF-2 (Fibroblast Growth Factor 2) and ELISA kit for FGF-2 were used as received following the protocol given by the supplier (R&D Systems, Minneapolis, MI, USA). The assay was performed using a 96-well plate (Sarstedt, Nümbrecht, Germany), and optical density (OD) was measured on a plate reader (Synergy HT, Bio Tek, Winooski, VT, USA). FGF-2 (Fibroblast Growth Factor 2) was purchased from the R&D System (Germany) and the fertilized eggs were obtained from LSL Rhein-Main GmbH (Dieburg, Germany). Pictures were taken with an upright microscope SMZ-168 equipped with a Moticam 2300 (Motic Europe, Barcelona, Spain).

### 2.2. Synthesis of Carboxylated Agarose (CA)

Native agarose (NA) (1 g) was transferred into a 3-necked round bottom flask reactor, equipped with a mechanical stirrer and pH meter. The agarose was dissolved in water at a concentration of 1% *w*/*v*. The reactor was cooled to 0 °C under vigorous mechanical stirring to prevent the agarose from gelling and then charged with TEMPO (0.160 mmol, 20.6 mg), NaBr (0.9 mmol, 0.1 g), and NaOCl (2.5 mL, 15% solution). During the reaction, the pH of the solution was adjusted to a pH of 10.8, and the degree of carboxylation was controlled by the dropwise addition of NaOH solution in different volumes. In the end, NaBH4 (0.1 g) was added to quench the reaction, and the solution was acidified to pH 8 and then stirred for an additional 1 h. The modified agarose was precipitated by the sequential addition of NaCl (0.2 mol, 12 g) and ethanol (500 mL). The product was collected by vacuum filtration using a fritted glass funnel and then extracted using ethanol. The ethanol was removed by extensive dialysis against water with changes every 12 h, for 2 days. The modified agarose was then freeze-dried overnight to yield a white fibrous fluffy solid.

### 2.3. Synthesis of Sulfated Agarose (SA)

Pyridine (20 mL) was transferred into a round bottom flask and then cooled to 4 °C using an ice bath. Chlorosulfonic acid (2 mL) was slowly added to the pyridine solution, after which, agarose (1 g) swollen in formamide (70 mL) for 1 h was added to the pyridine-chlorosulfonic acid solution. The mixture was then heated to 85 °C and stirred for 4 h. The reaction was quenched with NaOH (2 M) and sulfated agarose was precipitated in ethanol. The precipitated solid was isolated by filtration and washed with ethanol followed by dialysis for 2 days against deionized water using a cellulose acetate membrane with a molecular weight cut-off of 12–14 kDa. The sulfated agarose was freeze-dried for characterization.

### 2.4. Synthesis of Phosphorylated Agarose (PA)

Urea (7.5 mg) was dissolved in dry DMF (7.5 mL) at 95 °C under an inert atmosphere until a clear solution was obtained. Agarose (200 mg) was added to the solution and dissolved at 120 °C under argon. After the total dissolution of the agarose, 85% *v*/*v* concentrate phosphoric acid (1.25 mL) was added and then stirred for 3 h under reflux in an argon atmosphere. The reaction mixture was then cooled to room temperature and quenched with methanol (100 mL). The white powder so obtained was washed 3 times with methanol (50 mL) and filtered using a glass frit funnel. The modified agarose was then dialyzed for 2 days against deionized water using a cellulose acetate membrane with a molecular weight cut-off of 3.5 kDa. Finally, the solution was freeze-dried to yield a white powder which was further characterized.

### 2.5. Fourier-Transformed Infra-Red Spectroscopy (FTIR)

KBr pellet for FTIR was prepared as follows: Briefly, sample (2 mg) and KBr (200 mg) were milled using a pestle and mortar and then pressed for 10 min into discs using a 10-ton press. FTIR spectra were recorded on a Vector 22 instrument (Bruker Optics, Germany) and the Opus software, Version7 provided by the manufacturer was used to analyze and import the spectrum.

### 2.6. Molecular Dynamic (MD) Simulations

MD simulation was performed using the Desmond package from D. E. Shaw Research Lab (New York, NY, USA) used in Maestro version 8.5 from Schrödinger (Cambridge, MA, USA). Initial conformation was obtained from the x-ray structure of the agarose that was downloaded from the PDB library. Modified agarose was drawn from the PDB file directly inside the Maestro software. An implicit water model was built using the Desmond tool, resulting in a 10 Å square box build by following the TIP3P (transferable intermolecular potential 3 point) solution model. The simulations were run in an isothermal-isobaric model at 300 kPa at atmospheric pressure for 15 ns. Analyses of the results were performed using the tools available in the standard package of the VMD software from the Theoretical and Computational Biophysics Group of the University of Illinois (Champaign, IL, USA).

### 2.7. Electronic Circular Dichroism (CD)

CD spectra were obtained using a spectropolarimeter J-810 equipped with a Pelletier temperature cell PFD-425S from Jasco (Easton, MA, USA). A solution of 0.15% *w*/*v* of agarose (or modified agarose) was prepared in water by heating the sample to 90 °C for 15 min and then cooled down to 5 °C in the spectroscope chamber for 30 min before the measurement. Each spectrum is an average of three scans, and each spectrum of a given modification is a mean of three different batches of the material.

### 2.8. Zeta Potential

C Zeta potential was measured on a Delsa Nano C particle analyzer from Beckman Coulter (Germany). Agarose was dissolved in water at 90 °C for 15 min to obtain a 0.15% *w*/*v* solution that was cooled down at room temperature to ambient conditions one day prior to measurement. The measurement was performed at 5 °C and samples were equilibrated for 30 min before measurement. The flow cell was aligned to the laser prior to each measurement., and the measurement was repeated three times (n = 3) and an average was calculated with the value reported for a given modification being the mean of three different batches (total n = 9).

### 2.9. Rheology

The 2% *w*/*v* gels were melted and cast in a 6-well plate and allowed to solidify at room temperature. A circular gel sample in the size of the rheometer tool was punched out and used to measure the shear modulus. For the frequency sweep measurements, the sample was heated to 37 °C, followed by equilibration for 30 min before recording G′ and G″ with increasing rotation frequency from 0.01 rad/s up to 10 rad/s with a 1% deformation. The G′ of the gel was determined at 1 Hz shear frequency.

### 2.10. Environmental Scanning Electron Microscopy (ESEM)

Scanning Electron micrographs were obtained on an ESEM Quanta 250 FEG (FEI, Hillsboro, OR, USA). Samples of 2% *w*/*v* gels were prepared and 2 mL of this solution was freeze-dried for 24 h under 0.1 mbar vacuum in a 5 mL glass vial. The sample was vertically cut, and the interior of the sample was imaged at different magnifications. Images shown here are representative of different areas of several batches of a given sample.

### 2.11. Enzyme-Linked Immunosorbent Assay (ELISA)

Human Fibroblast Growth Factor (hFGF) was loaded in the 2% *w*/*v* hydrogels formulations prepared at a 1:1 weight ratio (SA:NA, PA:NA, CA:NA, and only NA) at a concentration of 5 µg/mL. The hydrogels were cast in a 96-well plate and PBS buffer was dispensed on top of the hydrogels. The plates were incubated for up to 1 week at 37 °C in a cell incubator. At defined time points, half of the incubation media was pipetted and replenished by an equal volume of fresh PBS buffer, and the pipetted volume was frozen for ELISA. The amount of FGF-2 released at each given time point was measured with an ELISA kit according to the supplier protocol. Optical density was measured at 450 nm with a correction wavelength set at 570 nm.

### 2.12. Chorioallantoic Membrane Assay (CAM Assay)

Fertilized eggs were incubated until day 3, and a window was cut open on the shell. The agarose(s) and FGF-2 were formulated in PBS and then drop cast onto a 180 mm square NY8H (nylon 8) sterile filter paper from Millipore (Darmstadt, Germany) and allowed to gel on a sterile Parafilm (Bemis (Amcor), Neenah, WI, USA). The hydrogels were then placed on the CAM of the fertilized egg on day 10 after the beginning of the incubation [35,36]. The eggs were then observed on an upright microscope, and pictures were taken. The number of blood vessels was counted manually using the following criteria: a blood vessel that reached the sample was considered a positive vascular structure. Each data point represents the mean of at least two gels incubated in two different eggs (n = 4).

### 2.13. Statistical Analysis

The cumulative release data were analyzed for statistical significance using the student’s *t*-test (TTEST package in Microsoft Excel software, V. 16) using a paired, 1-tailed distribution. A *p*-value of ≤ 0.05 was considered statistically significant (* (*p* ≤ 0.05), ** (*p* ≤ 0.01), *** (*p* ≤ 0.001) and **** (*p* ≤ 0.0001).

## 3. Results and Discussion

### 3.1. Sulfated and Phosphorylated Agarose

**Figure 1:**
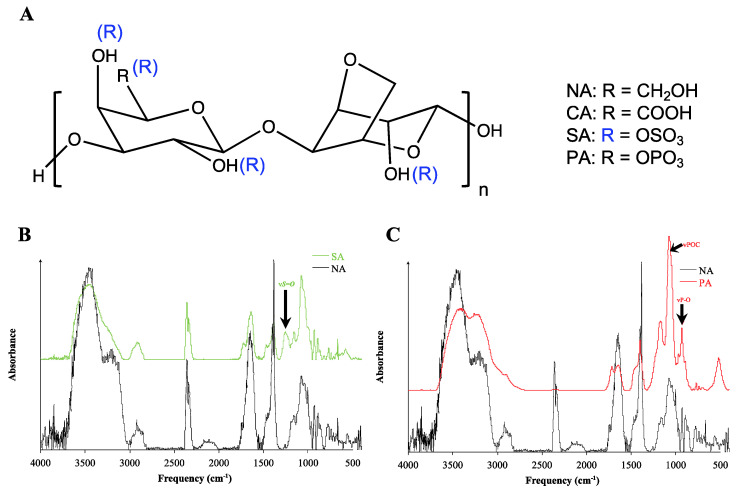
(**A**) Chemical structure of the repeat unit of native agarose (NA), carboxylated agarose (CA), sulfated agarose (SA), and phosphorylated agarose (PA). (**B**) Comparison of the FTIR absorbance spectra of NA and SA. The black arrow points to the vibration of S-O (ν_S = O_) in SA. (**C**) Comparison of the FTIR absorbance spectra of native agarose (NA) and phosphorylated agarose (PA). The black arrow points to vibrations of POC (ν_POC_) and PO (ν_PO_) in PA. The IR bands at 1158 and 1071 cm^−1^ correspond to –C–O–C– and glycosidic linkage [45], while the maxima around 1650 cm^−1^ is attributed to polymer-bound water [46].

**Figure A1:**
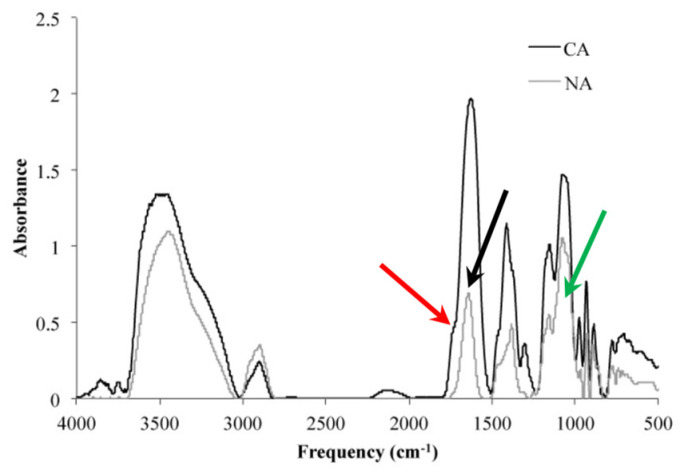
FTR spectra of native agarose (NA) and carboxylated agarose (CA). The green arrow indicates the absorption bands at 1158 and 1071 cm^−1^ corresponding to –C–O–C– and glycosidic linkage, the peak at 1650 cm^−1^ denoted by the black arrow denotes polymer-bound water, and the shoulder at 1750 cm^−1^ denoted by the red arrow corresponds to the carbonyl stretching in the carboxylic acid moieties in CA.

In ECM polysaccharides, post-functionalization with carboxyl, sulfate, and phosphate groups occurs mainly on the C6 position of the D-galactose or Xylose repeat unit [31,33,37]. To simulate such functionalization in a synthetic environment, the C6 position of the D-galactose repetition unit of native agarose (NA) was modified (Figure 1A). Under the reaction conditions employed, carboxylation and phosphorylation will occur primarily at the C6 position (hydroxymethyl) [24,38]. The sulfonation on the other hand can occur both at the equatorial and axial hydroxy groups, resulting in mono, di, or tri sulfonated agarose [39] (Figure 1A) The sulfated agarose (SA) synthesis was adapted from the literature, and the phosphorylated agarose (PA) synthesis was adapted from a synthetic route used to modify cellulose [40,41,42,43,44]. The 93% modified carboxylated agarose (CA) was synthesized by mild oxidation as described previously [24]. The modification was characterized by Fourier transform infrared (FTIR) spectroscopy. The IR spectra of NA in Appendix A Figure A1. The IR bands at 1158 and 1071 cm^−1^ in NA correspond to –C–O–C– and glycosidic linkage [45]. Since these bands are largely unaffected in CA (Appendix A Figure A1), SA, and PA (Figure 1B,C) it implies that the polymer backbone is preserved following backbone modification. Another prominent peak in the NA IR spectrum is a broad peak spanning 1750–1500 cm^−1^ with a maxima around 1650 cm^−1^. This peak can be attributed to polymer-bound water [46] since the IR spectrum was recorded under normal atmosphere and humidity this peak scales with the CO_2_ peak at 2250 cm^−1^, the changes in the peak height can be attributed to concentration. The FTIR spectrum of SA differs from that of NA in the presence of a peak at 1240 cm^−1^, corresponding to the vibration ν_S = O_. In the PA spectra, the bands at 1010 cm^−1^ and 900 cm^−1^ can be attributed to the ν_POC_ and ν_PO_, respectively.

### 3.2. SA and PA Polysaccharides Do Not Form Inter-Strand Bonds

**Figure A2:**
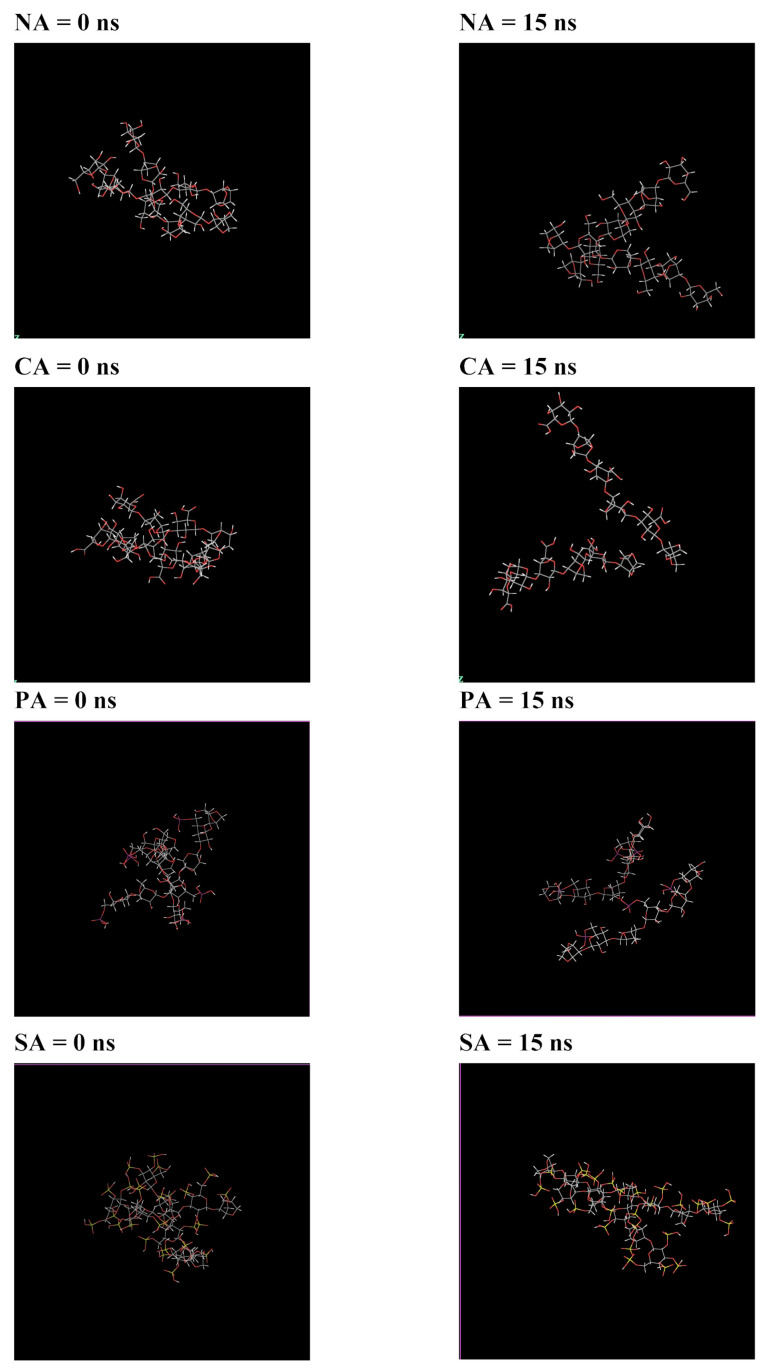
Visualization of the molecular dynamic simulation at the beginning of the experiment (t = 0 ns) compared to the conformation of the polysaccharide strands at the end of the experiment (t = 15 ns).

To understand the structural changes that are triggered by sulfation and phosphorylation MD simulations were carried out. It was observed that the conformation obtained at the end of the simulation is drastically different from CA (Appendix A Figure A2). This might imply that the incorporation of sulfate and phosphate moieties along the agarose backbone modifies the secondary structure of the polysaccharide strands. Indeed, the NA strands remain in contact during the whole simulation time, whereas the CA strands are separated at the end of the experiment. PA and SA also exhibit similar behavior, where only a limited interaction is observed between strands at the end of the simulation. It is also remarkable that where CA exhibits a stretch backbone, the SA and PA seem to conserve their helical conformation, which could be verified by circular dichroism (CD).

**Figure 2:**
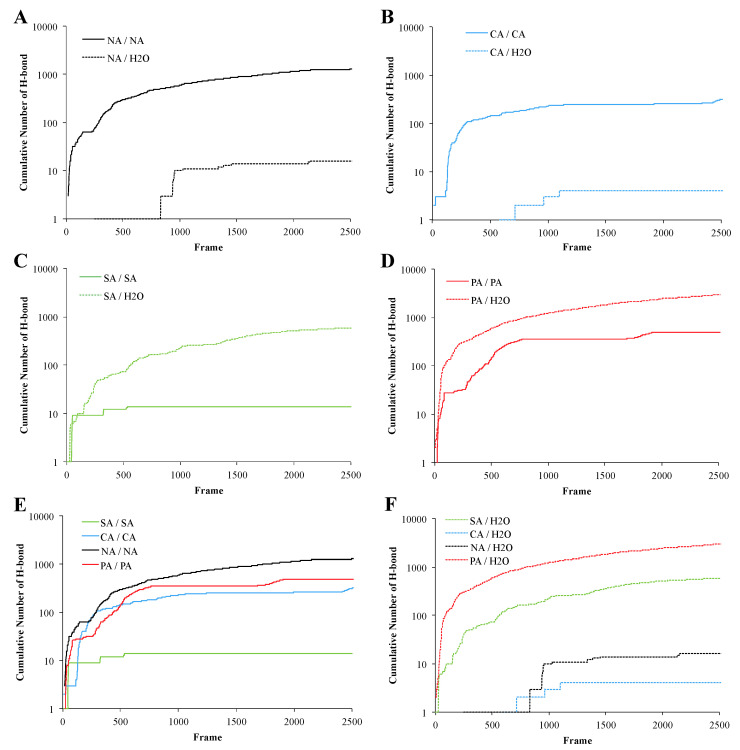
Cumulative H-bond number during the molecular dynamic simulation. Graphs (**A**–**D**) show a comparison between H-bonds calculated between two polysaccharide strands and the number of H-bonds between the polysaccharide strands and surrounding water molecules. Graphs (**E**,**F**) show a comparison between the interaction potential between strands of the modified agaroses in comparison to native agarose, and how these inter-strand interactions impact H-bonding between the strand and water. Simulations were carried out using the TIP3P water model.

The agarose hydrogel network is formed by α-helical strand–strand interactions through H-bonds [47]. The double helical regions agglomerate and lead to organized structures of higher order, which form the crosslink points [48]. To quantitatively assess the strand-strand interactions of the different polysaccharides and to understand the gelation mechanism of the newly synthesized SA, PA, and CA; the number of H-bonds formed between the strands during the simulation was compared to the H-bonds formed during the simulation between the polysaccharide strands and water molecule present (Figure 2). It was observed that NA and CA strands have a higher potential to form H-bonds with another strand than to form H-bonds with the surrounding water molecules, Figure 2A,B. On the other hand, the SA and PA polysaccharides were more likely to form H-bond with the surrounding water molecules than with their strands (Figure 2C,D). Furthermore, the PA-PA H-bond occurred at the same level as that in CA-CA and NA-NA even though the PA-H_2_O H-bond is higher (Figure 2E). Nevertheless, the strand-water H-bond interactions were of the same intensity for the CA and NA pair, and SA and PA pair (Figure 2F).

Based on these results, the aforementioned polysaccharides can be divided into two different classes: (1) agarose-based polysaccharides, which most likely form inter-strand H-bond (NA and CA) and (2) agarose-based polysaccharides, which most likely form H-bond with the surrounding water. This might imply that the CA and NA are in a lower hydration state as compared to the SA and PA polysaccharides. These findings were then substantiated by the characterization of the polysaccharide’s secondary structure.

### 3.3. SA and PA Exhibit Secondary Structures Similar to That of NA

**Figure 3:**
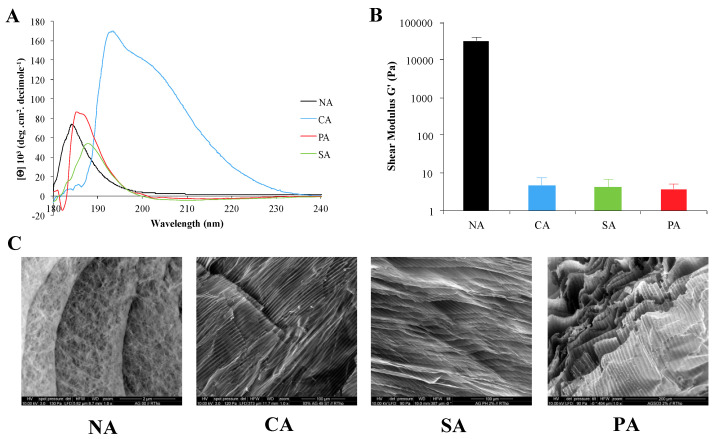
(**A**) Circular dichroism (CD) of 0.1% *w*/*v* solution of native agarose (NA), carboxylated agarose (CA), phosphorylated agarose (PA), and sulfonated agarose (SA). (**B**) Shear modulus (G′) of 2% *w*/*v* hydrogels of native agarose (NA), carboxylated agarose (CA), phosphorylated agarose (PA), and sulfated agarose (SA). (**C**) Scanning electron micrographs of freeze-dried gels prepared from 2% *w*/*v* solutions.

The secondary structures of the SA and PA strands were characterized by CD and compared to ones obtained for NA and CA (Figure 3A). The maximum ellipticity values for PA and SA were observed at 186 nm and 189 nm, respectively. However, the peak observed for the CA in the β-sheet region was not observed for the PA and SA. This reveals that the functionalization of agarose with carboxylic moieties uniquely induces an α-helical to β-sheet shift, while sulfate and phosphorylated agarose retain their α-helical structure. These measurements correlate well with the results of the MD simulation, where the conservation of α-helical organization upon backbone modification was predicted for both the SA and PA.

### 3.4. SA and PA Hydrogels Exhibit Similar Mechanical Properties to CA Hydrogels

Since the α-helical secondary structure in the NA polysaccharide is responsible for the formation of crosslinks in the hydrogel, one could assume that the conservation of the α-helices in SA and PA would provide hydrogels of similar stiffness as NA. However, the introduction of phosphate and sulfate groups diminished the shear modulus (G′) by three orders of magnitude compared to NA. But interestingly, the G′ values of SA and PA (G′ about 5 kPa) are similar to CA, which is rich in β-sheet structures (Figure 3B). Thus, while the secondary structures of CA, SA, and PA are different, they still exhibit similar stiffness. This could be rationalized by the differences in polymer chain hydration (polymer strand-water interactions), which can be inferred from the MD simulations. Since the hydration of polymer molecules will be impacted by the charges along the polymer backbone, this highlights a role for electrostatics in the observed changes to gel stiffness. To further decipher the role of the sulfate and phosphate groups, the organization of polymer fibers in the hydrogels was investigated.

### 3.5. Modification of the Agarose Backbone Impacts the Hydrogel Fiber Organization

The organization of SA and PA fibers in freeze-dried hydrogels was evaluated using ESEM. Electron micrographs of 2% *w*/*v* freeze-dried hydrogels reveal a change in the fiber organization upon functionalization across the different polysaccharides (Figure 3B). It was observed that while NA exhibits a random organization of fibers, a ribbon-like organization is observed for PA and SA (Figure 3C). Furthermore, the morphologies of the ribbon-like structures are similar in CA, PA, and SA. This fiber organization could be related to the loss of hydrogel stiffness. Indeed, as the fibers align along a single direction, chain entanglement is diminished, and the polymers are more susceptible to shear deformation [49]. Therefore, the comparable G′ of CA, PA, and SA may be attributed to the unique organization of modified agarose strands.

### 3.6. Proposed Polysaccharide Strand Organization for SA and PA

MD studies demonstrated that CA, SA, and PA polysaccharides lack or exhibit lower α-helical chain-chain interactions, which in turn lowers hydrogel stiffness. Therefore, a lack of α-helical chain-chain interaction seems to be driving the mechanical properties of the hydrogel. Since in CA, a high H-bond interaction between the polysaccharide strands was observed, the gel formation mechanism of SA and PA should, therefore, be different. 

**Figure 4:**
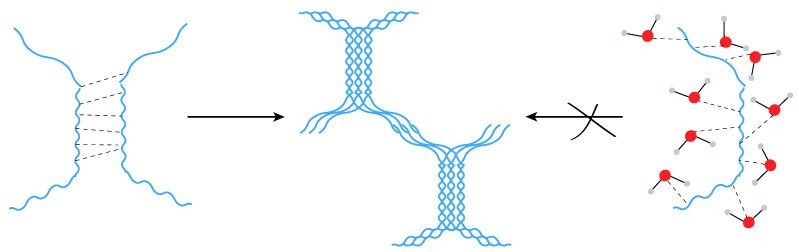
Mechanism of gelation of the NA (**left**) depicting the formation of helical bundles due to strong inter-strand H-bond in comparison to SA and PA (**right**) where the disruption of the helical structures lead to diminished polymer strand interactions and promote H-bonding with water molecules (red) resulting in weak gels.

The CD data revealed that strands of SA and PA retain their α-helical structure but, as predicted by MD simulations, fail to agglomerate with other helical strands; this leads to a soft hydrogel as confirmed by rheology. Therefore, we posit that the H-bond affinity of the polymer strands with water molecules competes with the H-bond formation between the polymer strands, thereby limiting the propensity of SA and PA to organize into a network. Based on this reasoning, the following gelation mechanism is proposed (Figure 4). While the α-helical ordered chains of the NA interact with each other through hydrogen bonding, the SA and PA chains fail to establish H-bonding with other polysaccharide strands, therefore restricting the formation of crosslinking points required for the formation of a hydrogel network. Nevertheless, the high affinity of SA and PA for water molecules provides a new hydration state for the polysaccharide strands, which could impact the thermodynamic activity of molecules present within this environment. This could be exploited to potentially control the uptake and release of biologically active soluble molecules.

### 3.7. Charge Characteristics of SA and PA

**Figure A3:**
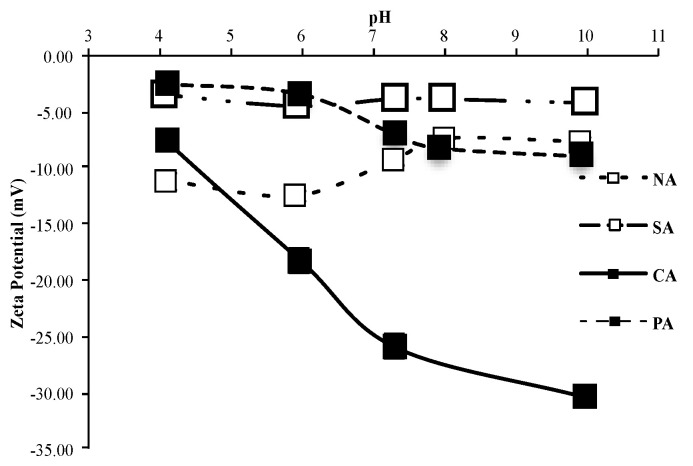
Zeta potential of the 0.1% *w*/*v* solution of native agarose (NA), carboxylated agarose (CA), phosphorylated agarose (PA), and sulfonated agarose (SA).

The next aim was to exploit SA and PA for controlled growth factor delivery. Since it is known that sulfated moieties of ECM polysaccharides, such as in heparin sulfate, are responsible for the immobilization of growth factor through electrostatic interactions, the zeta potential (ζ) of SA and PA was characterized. Interestingly, it was observed that the CA zeta potential (ζ= −28 mV) was lower than the one of SA, PA, and NA (ζ = −12 mV) (Appendix A Figure A3). Interaction of GF with sulfated moieties is observed in various synthetic and natural systems. Therefore, there should be some differences in the interaction of the FGF-2 with CA, and with the other formulations (SA, PA, NA.) This theory is further elaborated below.

### 3.8. Functionalization of Agarose Influences FGF-2 Release

**Figure 5:**
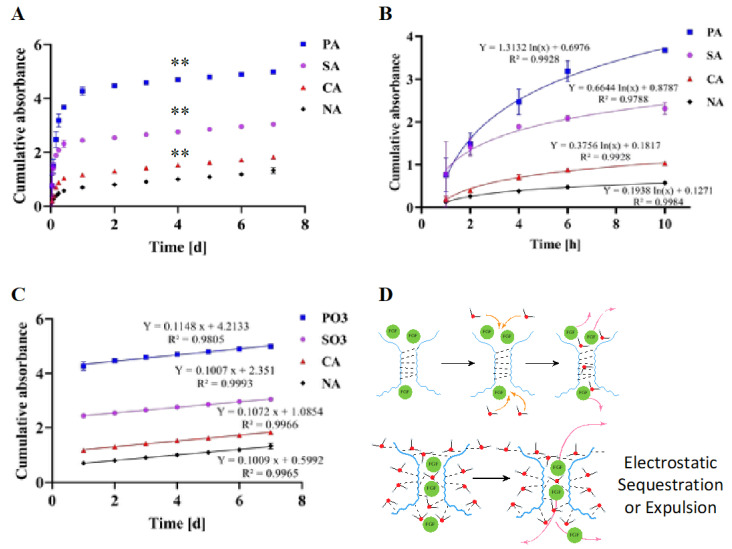
(**A**) FGF-2 release profile as measured by ELISA from the various modified agarose hydrogels (SA: sulfated agarose; PA: phosphate agarose, CA: carboxylated agarose) showing sustained release for a duration exceeding 1-week with appreciable differences in temporal and cumulative release in comparison to native agarose (NA) (** (*p* ≤ 0.01)). Data points represent average values with standard deviation (SD). SD bars are only visible in those that exceed size of the size of the symbol. One absorbance unit is 626 pg. All values beyond 10-h were found to be statistically significant with *p* values of ≤ 0.01 or ≤ 0.001 (see Figure A5 for *p*-values between various groups and time points) (**B**) Release profiles during the initial 10-h clearly showing the ability of these hydrogels to sequester FGF-2 to various degree. Release from PA beyond 2 h was found to be statistically significant, from all other conditions, and release from SA was statistically significantly different from CA and NA. (see Figure A5 for *p*-values between various groups and time points) (**C**) Release profile beyond 10 h showing linear behavior with similar slop from all hydrogels, suggesting that steady state release of FGF-2 is not impacted by charges characteristic of the hydrogels, implying that dissociation of the FGF-2 from polymer network is the limiting step. (**D**) The postulated release mechanism of FGF-2 from modified agarose hydrogels. In NA hydrogels (top cartoon) the dense polymer network due to strong interactions between agarose chain limits diffusion, while in the highly charged SA and PA hydrogels (bottom carton) electrostatic sequestration and expulsion dominate.

**Figure A4:**
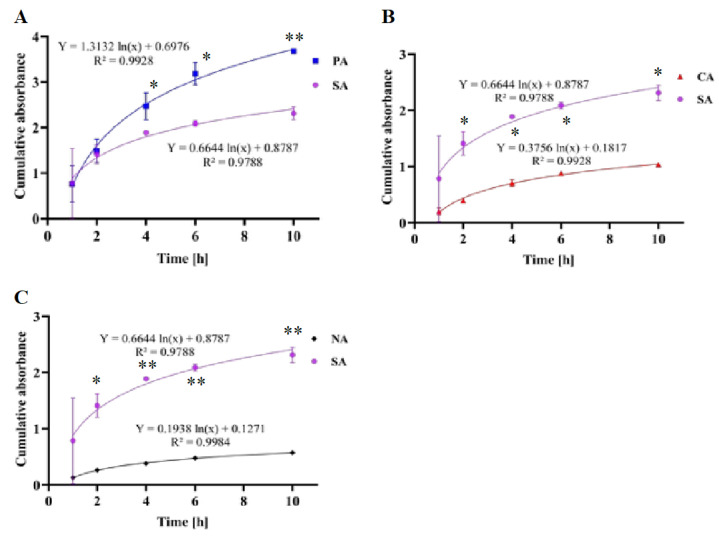
Paired comparison of the FGF-2 release profile over the first 10-h from SA (sulfated agarose) with that from PA (phosphate agarose) (**A**), CA (carboxylated agarose) (**B**), and NA (native agarose) (**C**) hydrogels as measured by ELISA. The plot shows cumulative absorbance as a function of time and the data points represent average values with standard deviation. (* *p* ≤ 0.05, ** *p* ≤ 0.01).

The delivery of growth factors involved in vascularization has been under extensive investigation for controlling sprouting angiogenesis, i.e., the formation of new blood vessels from the existing vasculature [50]. It is known that FGF-2 is responsible for the recruitment of endothelial cells and is of major importance in tumorigenesis and cardiovascular diseases [51,52]. Moreover, the short half-life of FGF-2 requires formulation strategies to optimize both the duration and concentration of release [53]. It is well established that naturally occurring ECM macromolecules bind to growth factors and serve as reservoirs. In addition to aiding in the hydration of the tissue, ECM macromolecules such as heparin and heparin sulfate are known to stabilize FGF and extend its mean half-life and biological activity [54,55]. For the ECM to act as a reservoir the GF needs to reversibly bind to the binding motifs in the ECM. Such interactions could be potentially achieved through protein-protein interactions [56,57], and/or electrostatic interactions [5]. The latter interactions are observed in sulfated polysaccharides such as heparin sulfate, dermatan sulfate, or chondroitin sulfate. With regard to the sustainable delivery of GFs, one could also modify existing polymers to reproduce such interactions. We propose that agarose polysaccharides bearing charged functional groups, such as carboxylate, sulfate, and phosphate along the backbone, can be used to control FGF release by modeling the native environment of the growth factors, such as binding and dissociation from ECM, the hydration state of protein–ECM complexes, and tissue stiffness. Since FGF interaction with sulfate groups is well established, the release behavior of FGF-2 from CA, PA, and NA gels was compared to the one from SA. To compensate for any loss in hydrogel stiffness over time and variation in stiffness and fiber organization between the various modified agaroses hydrogels were formed by mixing CA, SA, and PA with NA at a 1:1 ratio, thus ensuring that the gels had similar stiffness and thereby eliminating stiffness as a variable. The release study was carried out for 7 days and the release of FGF-2 was quantified by a conformation-sensitive enzyme-linked immunosorbent assay (ELISA) (Figure 5A). Cumulatively, FGF-2 release was ~2 times lower in the case of CA in comparison to SA gels, 4 times lower from NA as compared to SA, and about 1.8 times higher in the case of PA when compared to SA. The release of FGF-2 was, therefore, highest from PA gels, followed by SA and CA, with FGF-2 release from NA gels being the slowest, i.e., NA < CA < SA < PA. However, these differences can be accounted for by the release behavior in the first 10 h. A comparison of the first ten hours of the FGF-2 release revealed marked differences in the release profile depending on the charge characteristics in the hydrogel formulation (Figure 5B and Figure A4A–C). Since the initial loading concentration of the FGF-2 is identical in all cases, this suggests that FGF-2 has a different binding affinity to the polymer matrix within the various hydrogels, and this accounts for the differences observed in the cumulative release over time. That is, the steady-state release is likely governed by the diffusion of the FGF in the water phase of the gel aqueous network. 

### 3.9. Postulated Mechanism for FGF-2 Release

Based on the data at hand, we postulate two potential cooperative mechanisms, one involving FGF-2 interaction with the polymer matrix and the other involving the charged state of the hydrogel (Figure 5D). It has been shown in modeling studies that FGF-2 release during the first hour from hydrogel matrices bearing heparin electrostatically assembled in the hydrogel through a bidomain peptide is primarily due to dissociation of the complex rather than passive release of unbound FGF-2 [58]. So, it is reasonable to assume that the differences in release pattern observed in this study can be partly attributed to the differences in the interaction of FGF-2 (fraction bound) to the charged polymer backbone. Another mechanism could involve how charged hydrogels may impact the diffusion of macromolecules. We have recently shown that fixed negative charges in hydrogels can be exploited for controlled short-term release of proteins via a mechanism involving sequestration (+ve → ← -ve) and expulsion (-ve ← → -ve) based on the zeta potential of proteins [22]. The phosphate ester group has −2 negative charges at physiological pH (pKa 1.5, 6.3) [59], the sulfate group is completely ionized (>90%) as the pKa organo-sulfate monoester is expected to be below 1 [60], and the carboxylate group (pKa of 3.4) is partially ionized. Therefore, the gels have various degrees of fixed negative charges. Since FGF-2 has a net positive charge at physiological pH (isoelectric point of 9.49) [22], it should show strong associative tendencies with the polysaccharide matrix particularly in the PA and SA environment. Therefore, the higher release rate of FGF-2 from SA and PA matrices can be reconciled based on differences in the affinity for the FGF-2 to these functional groups. We posit that in the SA matrices, FGF-2 shows natural affinity to the sulfated polymer, which could be lacking in the PA system. However, the poor release from NA and CA could only be explained based on differences in the hydration status of the polymer network. One can infer from the MD simulations that SA and PA polysaccharides exhibit higher H-bond affinity with water than with their respective polysaccharide strands (Figure 2F). Additionally, the CA and NA polysaccharides exhibit higher H-bond affinity with themselves than water molecules. This trend is mirrored in the FGF-2 release, suggesting a role for polymer chain hydration in the release of the FGF-2. Since the steady-state release is governed by passive diffusion, stronger hydration of polysaccharides would favor higher FGF-2 diffusion into the aqueous network. It is noteworthy that after the first day, the FGF-2 release from all the gels exhibited a similar slope, which is consistent with the hypothesis that the associative interactions and dissociation of the FGF play a prominent role only earlier on and that the steady-state diffusion via aqueous pathways is independent of gel physicochemical characteristics (Figure 5C). Since blending each modified polysaccharide with the NA ensured that the hydrogel stiffness differences were minimized, stiffness could therefore be ruled out as a factor in the observed release behavior. Taken together, modified agaroses offer a potential platform for designing release systems for FGF-2 and other growth factors that are tailored to match the natural GF concentration.

### 3.10. The FGF-2 Released Is Biologically Active, Induces Angiogenesis in a CAM Assay, and Shows Dependence on Agarose Modification

**Figure 6:**
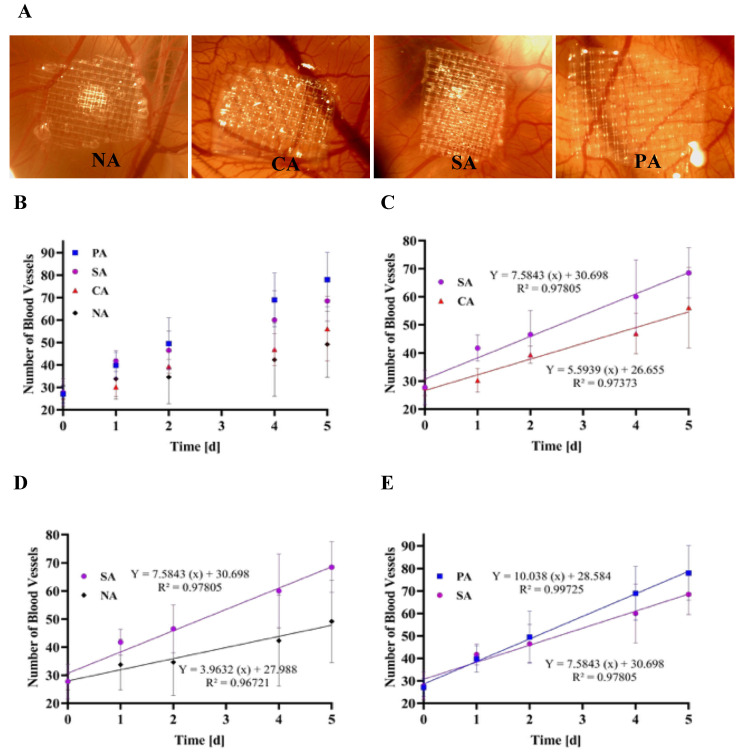
(**A**) Photographs of vasculature around the different hydrogel on the chorioallantoic membrane (CAM) of a chick egg. (**B**) Quantification of blood vessels formed around the different hydrogels (native agarose (NA), carboxylated agarose (CA), phosphorylated agarose (PA), and sulfonated agarose (SA)) loaded with FGF-2 and positioned on the CAM over a gelatin mesh. (**C**–**E**) Temporal changes in blood vessel numbers showing a higher propensity around SA hydrogels loaded with FGF-2 in comparison to CA and NA, and a similar propensity in comparison to PA hydrogels despite a higher release of FGF-2 from PA hydrogels, suggesting a possible role for sulfonate groups in SA hydrogel in stabilizing FGF-2.

In order to have clinical relevance FGF-2 has to be released in its active form. To assess the biological activity of the FGF-2 released from modified agarose-containing matrices, the chick chorioallantoic membrane (CAM) assay was used. The CAM assay has been extensively explored in cancer research as a reliable system for studying angiogenesis [36,61]. FGF-loaded hydrogels were placed on the CAM [35] of a fertilized egg and the number of blood vessels growing toward the FGF-2 gradient over 5 days was counted (Figure 6). It was observed that the blood vessels actively migrated toward the loaded hydrogels, thereby indicating that the released FGF-2 was biologically active with a linear increase in blood vessel induction and recruitment observed over 5 days. Furthermore, it was observed that the increase of the blood vessels is constant over time. This is of particular importance, as the formation of stable and mature blood vessels requires a steady growth factor delivery [36]. More importantly, the CAM assay confirmed the differences observed in vitro with the ELISA. While comparable amounts of blood vessel(s) (bv) were observed at the onset of the experiment, over time the vessel numbers increased at a rate of 3.6 and 3.9 bv/day for CA and NA, respectively, which was however, lower than the 7.5 bv/day and 10 bv/day observed in response to SA and PA hydrogels, respectively Figure 6A–C. These bv growth behaviors followed similar trends as the in vitro experiment where the PA exhibited the highest levels of FGF. Moreover, the two different hydrogel groups identified earlier as (1) a high hydration state (SA and PA) and (2) a low hydration state (NA and CA) are also recognized in this experiment, with NA and CA gels exhibiting lower bv growth kinetics in comparison to SA, and PA hydrogels. 

## 4. Conclusions

In conclusion, the results of this study support the potential of the SA, PA, and CA hydrogels as matrices for the delivery of growth factors. From a materials point of view, this study has helped decipher the role of the backbone modifications on the organization of the agarose polysaccharide chain. The introduction of sulfate and phosphate functional groups on the agarose backbone, while preserving the α-helical secondary structure of the polymer strands prevents the strands from forming hydrogen bonds with each other, thus favoring interaction with surrounding water molecules. This affinity for water molecules impacts the hydration state of the hydrogel, and its ability to form crosslinking points and thereby yield soft hydrogels. The classification of the modified agarose into two categories—(1) polymer–polymer H-bond and (2) polymer–water H-bond—has enabled the identification of two FGF release behaviors. While the first hydrogel category can favor slow diffusion of FGF albeit at low concentrations, the second favors a faster release. These results pave the way toward a system for sustained and controlled release of FGF in biological systems. Such injectable systems could be desirable in the management of chronic vascular disease and restoration of vasculature in diseased tissue sites.

## Figures and Tables

**Figure A5:**
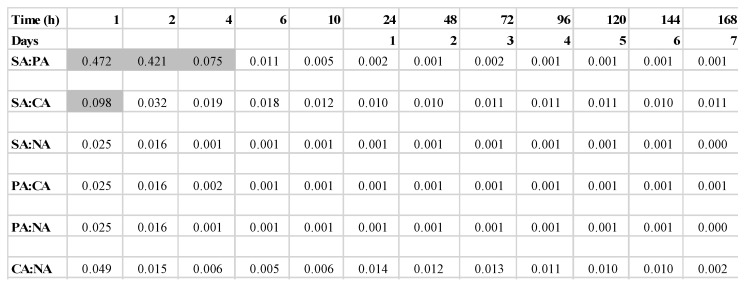
*p*-values for the cumulative release of FGF-2 at various time points between the various hydrogel groups. Significance was assessed using a student’s *t*-test (paired, one-tailed). A *p*-value of ≤0.05 was considered statistically significant (* (*p* ≤ 0.05), ** (*p* ≤ 0.01), *** (*p* ≤ 0.001) and **** (*p* ≤ 0.0001). Gray boxes represent comparisons that were not statistically significant.

## Data Availability

The original contributions presented in this study are included in the article; further inquiries can be directed to the corresponding author.
